# The Effect of Position Changing on Endotracheal Tube Cuff Pressure and Post-Operation Sore Throat and Hoarseness in Patients Undergoing General Anesthesia

**DOI:** 10.22038/ijorl.2025.85571.3870

**Published:** 2025

**Authors:** Masoomeh Tabari, Faezeh Rajabi, Ali Moradi, Alireza Sharifian Attar

**Affiliations:** 1 *Department of Anesthesiology, Faculty of Medicine, Mashhad University of Medical Sciences, Mashhad, Iran.*; 2 *Clinical Research Development Unit, Ghaem Hospital, Mashhad University of Medical Sciences, Mashhad, Iran.*; 3 *Orthopedic Research Center, Mashhad University of Medical Sciences, Mashhad, Iran.*

**Keywords:** Endotracheal tube, Cuff pressure, position, Hoarseness, Sore Throat

## Abstract

**Introduction:**

Endotracheal intubation is a standard procedure for securing and maintaining the airway during general anesthesia. Cuff pressure must be within the correct range to avoid serious airway complications. This study aimed to assess how the pressure in the endotracheal tube cuff changes when the patient’s position is altered.

**Materials and Methods:**

This prospective, observational study was conducted on 85 patients aged 18 to 75 undergoing general anesthesia for surgery. Endotracheal intubation was performed with an appropriately sized tube, and the tube cuff was inflated with air using a syringe. The cuff pressure of the endotracheal tube was then assessed using a cuff manometer immediately after intubation and position change, 5 minutes after each, and every 15 minutes until the end of the surgery. Based on the formula for testing the difference between two means for a quantitative trait in two populations, and considering an alpha of 0.05 and a beta of 0.2, the sample size was calculated as 20 individuals in each group of patients with different positions.

**Results:**

The endotracheal cuff pressure increased in all three positions, including prone, right lateral, and left lateral. A significant relationship was also observed between the sore throat one hour after extubation and the prone position.

**Conclusion:**

The ETT cuff pressure increased or decreased outside the normal range in most patients undergoing surgeries that require changing positions. Therefore, we recommend close and continuous monitoring of cuff pressure during anesthesia.

## Introduction

Endotracheal intubation is a standard procedure for securing and maintaining the airway during general anesthesia. The ideal endotracheal tube (ETT) cuff pressure is between 20 and 30 cmH2O (1).

Over-inflation of the endotracheal cuff causes the pressure inside the cuff to surpass the capillary perfusion pressure of the tracheal mucosa (2). 

Tracheal hypoperfusion is associated with ischemia, stenosis, necrosis, ulceration, fistula, and respiratory complications such as cough, sore throat, and hoarseness (2,3). However, when the cuff pressure is too low, secretions may be inhaled into the lungs, resulting in ventilator-associated pneumonia (4-6). The ETT cuff pressure is typically checked once right after intubation by palpating the pilot balloon or using a manometer (4). However, the ETT cuff pressure can change later due to factors like airway pressure and the patient's position during surgery (5, 6). 

Given the importance of the mentioned complications, regular monitoring of the endotracheal tube cuff pressure is essential.Some surgical procedures, such as nephrectomy and PCNL, involve adjusting the patient's position. The pressure on the ETT has been reported to be higher in the lateral compared to the neutral position (7). It has also been reported that changing from the supine to the prone position in lumbar spine surgeries alters the cuff pressure (8, 9). 

As a result, in surgeries that involve position changes, especially when the surgery times are long, the cuff pressure may be subjected to significant changes. According to the reviews, comprehensive studies examining cuff pressure at various positions and postoperative complications are lacking.

We aimed to investigate the association between endotracheal tube cuff pressure, patient positions during surgery (prone, right and left lateral, and supine), and post-extubation complications, including hoarseness and sore throat, in patients undergoing urological, orthopedic, and general surgeries.

## Materials and Methods

This prospective, double-center, observational study was conducted at Ghaem and the Imamreza Hospital, Mashhad University of Medical Sciences, Mashhad, Iran. The study was approved by the University ethics committee (IR.MUMS.IRH.REC.1402.237). A written informed consent was obtained from all the patient participants.

Eighty-five Patients aged 18 to 75 years undergoing urological, orthopedic, and general surgeries, in which the patient's position changed during the procedure, were evaluated. Each patient was initially monitored supinely using electrocardiography, blood pressure, and pulse oximetry. General anesthesia was then induced using a preoxygenation protocol with 100% oxygen, Midazolam, Sufentanil or Fentanyl, Propofol, and Cisatracurium. 

Endotracheal intubation was performed with an ETT sized 6.5/7 for females and 7.5/8 for males, and the tube cuff was inflated with air using a syringe. The ventilator was set to a volume-controlled mode with a tidal volume of 6 cc/kg and a PEEP of 5 cmH2O. The ETT cuff pressure was assessed using a cuff manometer (VBM cuff pressure gauge with hook) immediately after intubation, position change, 5 minutes after each, and every 15 minutes until the end of the surgery. 

Although the ETT cuff pressure was constant during the operations, it was re-adjusted whenever it exceeded the normal range (20-30 cm H₂O). The sample size was calculated according to the outcomes of a similar study and considering an α=0.05 and a β+0.2, using the formula for comparing the means in two populations (8). The patients were also evaluated for hoarseness, sore throat, and cough an hour after the surgery. The patients scored sore throat using the visual analogue scale (VAS).

## Results

The data from 85 patients were finally analyzed. Table 1 presents the characteristics of the study population, including the age, gender, Body Mass Index, airway physical exams, past medical history, ASA (American Society of Anesthesiologists Class: a risk-stratifying system used to help predict preoperative risks) classes, and ETT size and type.

**Table 1:** Patient demographics in four groups with different head positions

		**Patient Head Position**				
		**Supine** **n=22**	**Right lateral** **n=20**	**Left lateral n=23**	**Prone** **n=20**	**P**
Age		50.59±13.376	50.85±18.123	49.74±15.431	47.7±12.427	0.907*
Gender	Male	12	9	10	11	0.811**
	Female	10	11	13	9	
Body Mass Index		25.04	31.56	26.8	25.84	0.661*
Mallampati Score	1	8	3	6	4	0.721**
	2	11	11	10	9	
	3	2	6	6	6	
	4	1	0	1	1	
Thyromental Distance	<6Cm	1	1	1	3	0.471**
	>6Cm	21	19	22	17	
Open Mouth	Good	22	18	22	18	0.441**
	Restricted	0	2	1	2	
Upper Lip Bite Test	OK	17	13	17	12	0.598**
	Not OK	5	7	6	8	
ASA class^1^	1	5	10	13	12	0.214**
	2	12	5	5	3	
	3	4	4	4	5	
	4	1	1	1	0	
Tube Type	Regular	22	17	21	0	<0.001**
	Armoured	0	3	2	20	
Tube Size	6.5	2	0	1	2	0.265**
	7	10	9	12	6	
	7.5	7	8	4	11	
	8	3	3	6	1	
Intubation Duration		18.86±7.55	15.25±5.73	18.26±5.35	26.25±23.95	0.053*
Operation Duration		113.18±26.07	170.5±54.82	176.1±34.21	137.25±56.6	<0.001*
Diabetes Mellitus		3	0	4	4	0.234**
Hypertension		5	6	7	3	0.622**
Hyperthyroidism		5	1	0	1	0.033**
Ischemic Heart Disease		2	2	0	3	0.337**

^1^American Society of Anesthesiologist Class: a risk-stratifying system used to help predict preoperative risks

**Fig 1:**
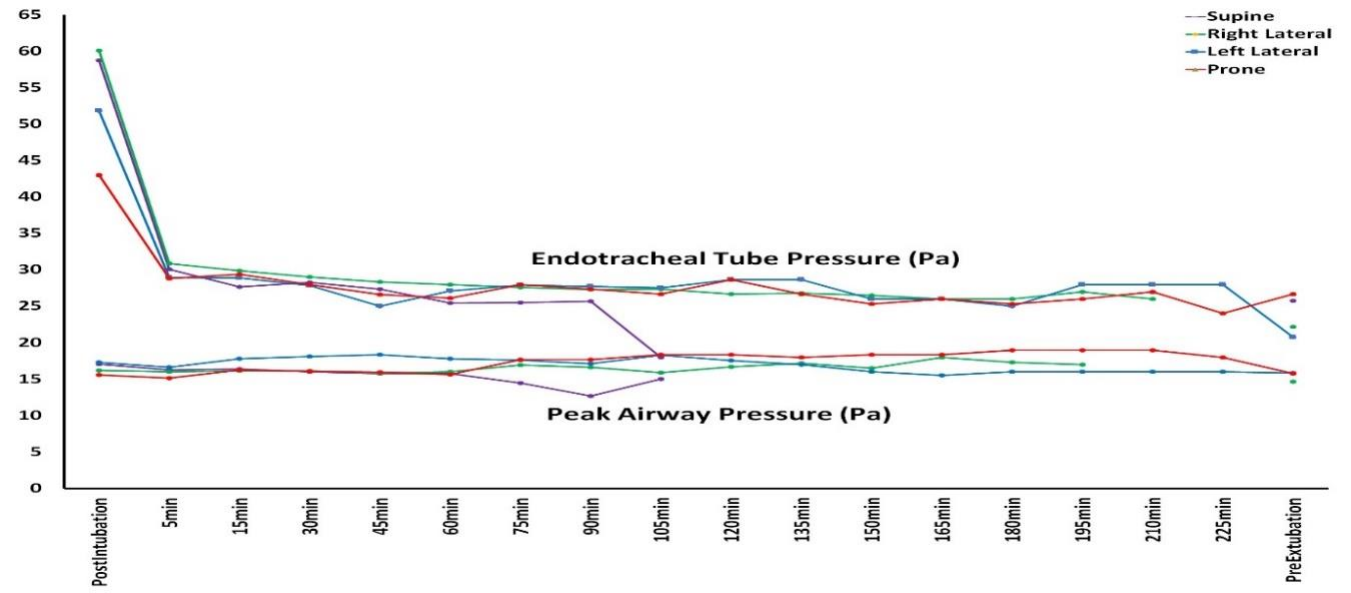
The trends for ETT cuff pressure and peak airway pressure from the post-intubation to pre-extubation.

Figure 1 shows the trends for ETT cuff pressure and peak airway pressure. The ETT cuff pressure in all four groups was significantly high in the supine position immediately after intubation, probably because of excessive tube inflation, but it remained almost constant during the operation. 

**Fig 2:**
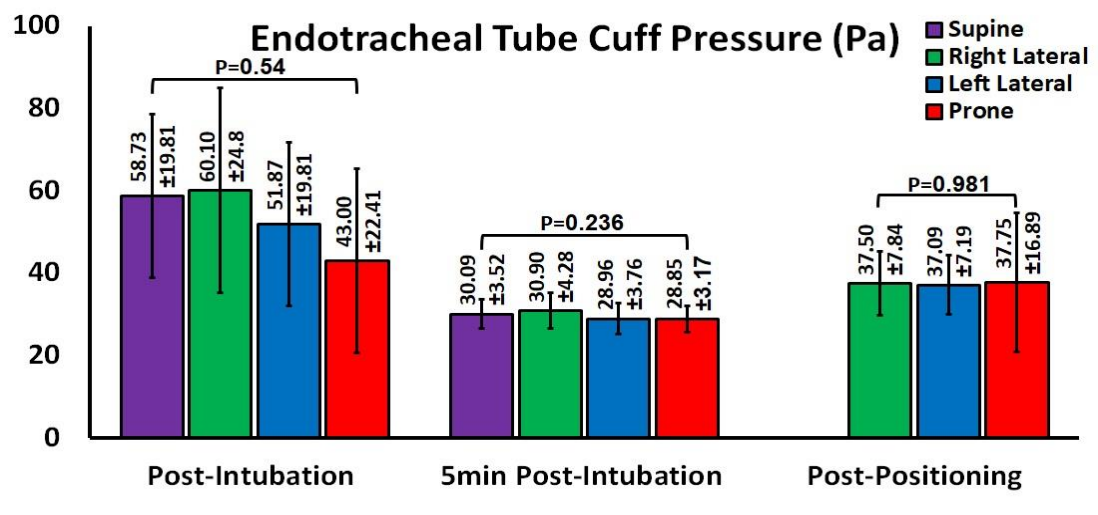
Endotracheal tube cuff pressure after intubation, five minutes later, and after changing position from supine to right lateral, left lateral, and prone position.

No significant statistical difference was seen in the ETT cuff pressure at four different positions (supine, right lateral, left lateral, and prone) exactly after intubation (P=0.54), 5 minutes later (P=0.236) (Figure 2). However, changing the patients' positions from supine to the right lateral, left lateral, and prone, resulted in significant increases in the ETT cuff pressures (P=0.0021, P=0001, and P=0.026, respectively).

**Fig 3:**
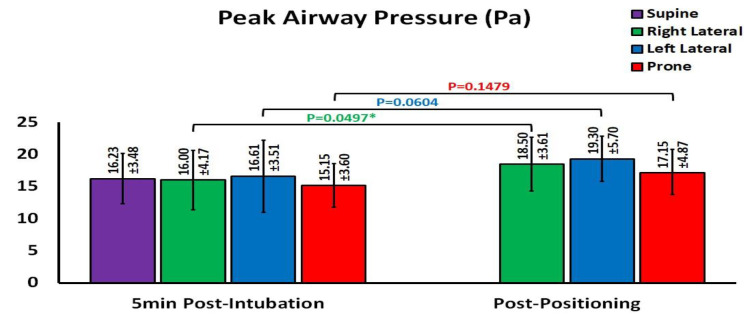
Peak airway pressure after intubation, five minutes later, and after changing position from supine to right lateral, left lateral, and prone position.

Also, no significant difference was seen in the peak airway pressure between different positions after intubation (P=0.56), five minutes later (P=0.624), and after changing the patient’s position (P=0.351) (Figure 3). However, the increases in peak airway pressures after positioning from supine to the right lateral, left lateral, and prone were not statistically significant (P=0.05, P=0.0604, and P=0.1479, respectively).

While no significant difference was found in the frequency of post-extubation hoarseness (P=0.847) and cough (P=0.971) between the four positions, a significantly higher frequency of patients (n=5(25%)) in the prone position experienced sore throat after extubation (P=0.038) (Table 2).

**Table 1:** The frequency of post-extubation sore throat, hoarseness, and cough in patients operated in different positions

	**Supine** **n=22**	**R.L.** **n=20**	**L.L.** **n=23**	**Prone** **n=20**	**P**
Sore Throat	1	0	2	5	0.038**
Hoarseness	1	2	1	1	0.847**
Cough	3	2	3	3	0.971**
VAS	1.32±1.49	1	1.43±1.47	2.45±2.6	0.042*

## Discussion

To elucidate the effect of patient surgical position on endotracheal tube cuff pressure and peak airway pressure as well as post-extubation cough, hoarseness, and sore throat, we studied 85 patients undergoing surgeries in four different positions, including the supine, right lateral, left lateral, and prone. Our findings showed statistically identical cuff and peak airway pressures between different positions, while the cuff pressures in all groups of patients increased after positioning from the supine. 

Also, except for the higher frequency of sore throat in patients operated in the prone position, the frequency of sore throat, hoarseness, and cough was homogeneous between the other positions.

A former study showed that the cuff pressure increased 47.3% and decreased 2.5% after changing the body position to right or left lateral (10). While these findings align with ours regarding the higher likelihood of an increase in cuff pressure rather than a decrease, they observed a larger proportion of subjects with increased and decreased cuff pressures. We observed that the ETT cuff pressure elevated beyond the normal range in most patients in the prone position. Along with this finding, another study also stated that the prone position increases the cuff pressure (8). 

It reported that the initial neutral pressure increased after changing position from supine to prone (26.0 vs. 31.5 ± 5.9 cmH2O, P<0.001) (8). Toshiyuki Minonishi et al. also stated that after the supine-to-prone position change, 91.7% of patients had ETT tube displacement, ETT cuff pressure decreased, and the ETT tended to withdraw (9). 

Our findings suggest that changes in position can potentially result in cuff pressure exceeding the normal range. As a result, we recommend monitoring the cuff pressure whenever the head and neck position is altered, particularly in the prone position. We also aimed to determine which head position change leads to the greatest variation in cuff pressure. Our findings indicated a significant association between the right lateral, left lateral, and prone positions and placement of the endotracheal tube at the patient's mouth corners. Increased ETT cuff pressure was also found after changing position from supine to right lateral, left lateral, and prone. A cuff pressure around 20-30 cm H2O is recommended to avoid cuff-related complications, such as ventilator-associated pneumonia and tracheal injury, in patients undergoing prolonged ventilation in the intensive care unit (11,12). 

The relatively high ETT cuff pressure during short surgical procedures can also lead to postoperative complications like sore throat, cough, and hoarseness (13). When the cuff pressure exceeds 30 cm H20, the blood supply to the tracheal mucosa decreases and causes injury (13,14). This study observed a significant association between the prone position and sore throat one hour after extubation. 

Our study had some limitations: First, the attending anesthesiologist could not be blinded to group assignment, which may be considered a source of bias. Second, the postoperative sore throat assessed in this study was based on subjective reports, and we did not investigate actual tracheal injury through direct visualization or histological examination. 

Third, pulmonary complications such as atelectasis, pneumonia, and hypoxemia were not assessed, so the relationship between intraoperative ETT cuff pressure and these complications is yet to be elucidated in future research. Finally, as this study was conducted solely in an Asian population, studying other ethnic groups is suggested.

## Conclusion

Declarations and According to the current study, changing the patient’s position from supine to any other position may result in fluctuations in ETT cuff pressure. Hence, close and continuous monitoring of ETT cuff pressure during anesthesia for long-lasting surgeries where the patient’s position is changed is necessary to avoid prolonged critical overinflation or underinflation. 

## Data Availability

The datasets generated during and/or analyzed during the current study are available from the corresponding author upon reasonable request.

## References

[1] Letvin A, Kremer P, Silver PC, Samih N, Reed-Watts P, Kollef MH (2018). Frequent Versus Infrequent Monitoring of Endotracheal Tube Cuff Pressures. Respir Care.

[2] Sultan P, Carvalho B, Rose BO, Cregg R (2011). Endotracheal tube cuff pressure monitoring: a review of the evidence. J Perioper Pract.

[3] Lizy C, Swinnen W, Labeau S, Poelaert J, Vogelaers D, Vandewoude K (2014). Cuff pressure of endotracheal tubes after changes in body position in critically ill patients treated with mechanical ventilation. Am J Crit Care.

[4] Lee J, Reynolds H, Pelecanos AM, van Zundert AA (2019). Bi-national survey of intraoperative cuff pressure monitoring of endotracheal tubes and supraglottic airway devices in operating theatres. Anaesth Intensive Care.

[5] Park JH, Lee HJ, Lee SH, Kim JS (2021). Changes in tapered endotracheal tube cuff pressure after changing position to hyperextension of neck: A randomized clinical trial. Medicine (Baltimore).

[6] Rosero EB, Ozayar E, Eslava-Schmalbach J, Minhajuddin A, Joshi GP (2018). Effects of Increasing Airway Pressures on the Pressure of the Endotracheal Tube Cuff During Pelvic Laparoscopic Surgery. Anesth Analg.

[7] Kim HC, Lee YH, Kim E, Oh EA, Jeon YT, Park HP (2015). Comparison of the endotracheal tube cuff pressure between a tapered- versus a cylindrical-shaped cuff after changing from the supine to the lateral flank position. Can J Anaesth.

[8] Kim D, Jeon B, Son JS, Lee JR, Ko S, Lim H (2015). The changes of endotracheal tube cuff pressure by the position changes from supine to prone and the flexion and extension of head. Korean J Anesthesiol.

[9] Minonishi T, Kinoshita H, Hirayama M, Kawahito S, Azma T, Hatakeyama N (2013). The supine-to-prone position change induces modification of endotracheal tube cuff pressure accompanied by tube displacement. J Clin Anesth.

[10] Okgun Alcan A, Yavuz van Giersbergen M, Dincarslan G, Hepcivici Z, Kaya E, Uyar M (2017). Effect of patient position on endotracheal cuff pressure in mechanically ventilated critically ill patients. Aust Crit Care.

[11] Mizuno S, Glowacki J (2005). Low oxygen tension enhances chondroinduction by demineralized bone matrix in human dermal fibroblasts in vitro. Cells Tissues Organs.

[12] Lorente L, Blot S, Rello J (2007). Evidence on measures for the prevention of ventilator-associated pneumonia. Eur Respir J.

[13] Liu J, Zhang X, Gong W, Li S, Wang F, Fu S (2010). Correlations between controlled endotracheal tube cuff pressure and postprocedural complications: a multicenter study. Anesth Analg.

[14] Brown BM, Oshita AK, Castellino RA (1983). CT assessment of the adult extrathoracic trachea. J Comput Assist Tomogr.

